# Identification of MicroRNAs in Response to Different Day Lengths in Soybean Using High-Throughput Sequencing and qRT-PCR

**DOI:** 10.1371/journal.pone.0132621

**Published:** 2015-07-10

**Authors:** Wenbin Li, Pengpeng Wang, Yongguang Li, Kexin Zhang, Fuquan Ding, Tengkun Nie, Xue Yang, Qingxue Lv, Lin Zhao

**Affiliations:** Key Laboratory of Soybean Biology of Chinese Education Ministry (Key Laboratory of Biology and Genetics & Breeding for Soybean in Northeast China), Northeast Agricultural University, Harbin 150030, China; Nanjing Agricultural University, CHINA

## Abstract

MicroRNAs (miRNAs) are short, non-coding single-strand RNA molecules that play important roles in plant growth, development and stress responses. Flowering time affects the seed yield and quality of soybean. However, the miRNAs involved in the regulation of flowering time in soybean have not been reported until recently. Here, high-throughput sequencing and qRT-PCR were used to identify miRNAs involved in soybean photoperiodic pathways. The first trifoliate leaves of soybean that receive the signal of light treatment were used to construct six libraries (0, 8, and 16 h under short-day (SD) treatment and 0, 8, and 16 h under long-day (LD) treatment). The libraries were sequenced using Illumina Solexa. A total of 318 known plant miRNAs belonging to 163 miRNA families and 81 novel predicted miRNAs were identified. Among these, 23 miRNAs at 0 h, 65 miRNAs at 8 h and 83 miRNAs at 16 h, including six novel predicted miRNAs at 8 h and six novel predicted miRNAs at 16 h, showed differences in abundance between LD and SD treatments. Furthermore, the results of GO and KEGG analyses indicated that most of the miRNA targets were transcription factors. Seven miRNAs at 0 h, 23 miRNAs (including four novel predicted miRNAs) at 8 h, 16 miRNAs (including one novel predicted miRNA) at 16 h and miRNA targets were selected for qRT-PCR analysis to assess the accuracy of the sequencing and target prediction. The results indicated that the expression patterns of the selected miRNAs and miRNA targets showed no differences between the qRT-PCR and sequencing results. In addition, 23 miRNAs at 0 h, 65 miRNAs at 8 h and 83 miRNAs at 16 h responded to day length changes in soybean, including six novel predicted miRNAs at 8 h and six novel predicted miRNAs at 16 h. These results provided an important molecular basis to understand the regulation of flowering time through photoperiodic pathways in soybean.

## Introduction

Environmental factors (day length and temperature) and internal signals (gibberellin and autonomous pathways) are integrated to regulate the flowering time of plants [1]. Higher plants recognize fluctuations in day length, which facilitate the coordination of the flowering time with changing seasons. To explain how photoperiodic organisms perceive day-length signals, the external coincidence model has been proposed [2]. Photoreceptors, circadian clock components, bio-clock and light regulated genes are key components for day-length detection in the external coincidence model. Among the clock- and light-regulated genes, *CO* has been identified as a key gene in the integration of light and clock signals. The overexpression of the *CO* gene leads to early flowering in Arabidopsis through the regulation of the expression of downstream genes, such as *FT*, *AP1* and *LFY*, regardless of the length of day light [1, 3–5].

MicroRNAs (miRNAs) are genomic DNAs encoded as ~20–22-nt small RNAs that function as endogenous regulators in eukaryotes. In plants, primary miRNAs (pri-miRNAs), which have a characteristic hairpin structure, are transcribed from MIR genes through polymerase II [6,7]. DCL1, a Dicer-like protein with a key role in the maturation of plant miRNA, makes the first two cuts, resulting in the conversion from pri-miRNAs into pre-miRNAs. DCL1 also facilitates the subsequent conversion of miRNA-miRNA* duplexes through HYPONASTICLEAVES 1 (HYL1) and SERRATE (SE). The duplex is exported into the cytoplasm through HASTY (HST1) [8]. Incorporated with AGO protein [9], miRNAs translocate to the RNA-induced silencing complex (RISC). In the RISC, miRNAs bind to complementary regions of messenger transcripts to guide mRNA degradation or translational repression, resulting in post-transcriptional gene silencing [10–13].

Since the first miRNA (*lin-4*) was identified in 1993 [14], 35,828 mature miRNAs have been recorded in 223 species in the current release (Release 21) of the miRBase [15–17]. Plant miRNAs play important roles in growth, development, stress responses and even the regulation of miRNA biogenesis [18,19]. In Arabidopsis, some miRNAs have been implicated in the regulation of flowering time through signal pathways, such as autonomous, gibberellins (GAs) and photoperiod [15,20–22]. MiR172 regulates plant flowering time through the photoperiod pathway [22,23]. It was reported that the *AP2-like* genes might be floral repressors downstream of *CO* and *FT*, but upstream of *LFY* [1]. The miR172 shared partial sequence complementarity with *AP2* and other *AP2-like* genes and had previously been shown to regulate the expression of the *AP2* gene during translation [24, 25]. Plant flowering time is also regulated by miR159 through the gibberellins (GAs) response pathways [21, 26]. The miR159 regulates the expression of *MYB* gene family members, many of which are expressed in the shoot apices and are essential for plant development [26]. *MYB* genes upstream of *LFY* in the gibberellin response pathways could bind to the promoter region of the *LFY* genes to promote flowering [21]. In the autonomous pathways, miR156 acts as a key negative regulator. MiR156 regulates the expression of the *SQUAMOSA PROMOTER BINDING-LIKE (SPL)* family of transcription factors and affects the phase transition from vegetative growth to reproductive growth [20, 27]. Transfering Arabidopsis from SD (short day) to LD (long day) conditions rapidly increased the transcription of the *SPL* transcription factor family and promoted flowering. Studies have reported that miR156 decreases with the increasing miR172 during plant growth [23]. In addition, *SPL* protein binds to the miR172 promoter to increase miR172 abundance and subsequently promote plant flowering [27]. Therefore, miR172 and miR156 might work together to regulate flowering time, although these molecules are involved in different pathways.

Soybean is one of the most economically important legume crops, providing both oil and protein for human consumption and animal forage. As a typical SD plant, soybean is sensitive to day length changes. The flowering time directly affects the output of soybean plants. Many reports have identified the miRNAs involved in growth, development, and stress responses in soybean. These studies have reported the identification of miRNAs that accumulate in response to biotic and abiotic stresses [28], aluminum [29] and phosphorus deficiencies [19], seed [30], shoot apical meristem [31] and nodule development [32, 33].

In the present study, both RNA sequencing and qRT-PCR were used to identify soybean miRNAs in response to the changes in day length. Known miRNAs and novel predicted miRNAs were identified and the functions of these molecules associated with the regulation of flowering time were evaluated. These findings provide a theoretical basis for improving the adaptation of soybean cultivars.

## Materials and Methods

### Plant materials and light treatments

The seeds of photoperiod-sensitive soybean cultivars ‘DongNong 42’ developed at Northeast Agricultural University in China were cultivated in growth chambers at 25°C under LD conditions (16-h light/8-h dark) with 250 μmol m^-2^sec^-1^ white light. The plants were transferred to SD conditions (8-h light/16-h dark) under the same temperature regime after the trifoliate leaves were expanded. The trifoliate leaves at 0, 8 and 16 h under both conditions at 20 d after emergence were collected and immediately frozen in liquid nitrogen and stored at -80°C. The samples were continuously collected for two days, and each sample comprised the leaves from three plants. The samples were mixed at the same time point from two days to reduce errors. A total of six small RNA libraries were constructed from RNAs under different light treatments at different sampling times: SD-0, SD-8, SD-16, LD-0, LD-8 and LD-16 h.

### RNA isolation, library construction and sequencing

Total RNA was isolated from fresh leaves using Trizol (TAKARA, Dalian, China). AGE (agarose gel electrophoresis) was used to evaluate the quality of the total RNA. The OD_260_ of the RNA was verified to ensure the amount of the RNA using a spectrophotometer (Eppendorf, Hamburg, Germany). Solexa/Illumina sequencing was performed at Biomarker Company (Beijing, China). The libraries were constructed from the six samples (SD-0, SD-8, SD-16, LD-0, LD-8, and LD-16 h). The main reagents and supplies were obtained from the Illumina Gene Expression Sample Prep kit and an Illumina Sequencing Chip (Flowcell), and the main instruments were the Illumina Cluster Station and the Illumina HiSeq 2000 System. Briefly, the RNA was purified using Oligo (dT) magnetic beads. Oligo (dT) was used as a primer to synthesize first and second-strand cDNA. The bead-bound cDNA was subsequently digested using a restriction enzyme at the CATG site. Illumina adaptor 1 was ligated to the sticky 5' end of the digested bead-bound cDNA fragments, and the restriction enzyme *Mme*I cut at 17-bp downstream of the CATG site. The Illumina adaptor 2 was ligated to the 3' ends of tags and acquired tags using different adaptors at both ends to form a tag library. After PCR amplification, purification and denaturation, the single-chain molecules were fixed onto the Illumina Sequencing Chip (Flowcell), and four types of nucleotides labeled with four different colors were added, followed by sequencing using the synthesis (SBS) method. Each line of the flowcell tunnel generated millions of raw reads with sequencing lengths of 35 bp [34].

### Prediction of conserved and novel predicted miRNAs

After removing the impure sequences (the low quality reads, adaptor reads and reads with length < 16 bp or length > 30 bp), the unique sequences were mapped to the *Glycine max* L. Merr. Cultivar ‘Williams 82’ genome (http://www.phytozome.net/) using the SOAP program. The perfectly matched reads were mapped to the Sanger Non-Coding RNA database (including tRNAs, rRNAs, snoRNAs, and other ncRNAs except microRNAs) using SOAP. After removing the non-coding RNA, the remaining unique sequences were subsequently mapped to the *Glycine max* microRNA precursors in the Sanger miRBase using SOAP to identify the known microRNAs. The criteria to define a non-coding RNA and a known microRNA were the same as previously reported [19]. The remaining unique sequences were subsequently used to predict the novel miRNAs. The upstream and downstream genomic sequences (200 bp each) of the sequence alignment position were extracted from the genome assembly using homemade Perl scripts and subsequently used to predict the pre-miRNA using the MIREAP program (http://sourceforge.net/projects/mireap). The secondary structure of the novel miRNA was predicted using RNAfold (http://rna.tbi.univie.ac.at/cgi-bin/RNAfold.cgi). The criteria to define a novel microRNA were the same as previously reported [35].

### Prediction of miRNA targets

The targets of known miRNAs and novel predicted miRNAs were predicted using the psRNATarget program (http://plantgrn.noble.org/psRNATarget), which identifies putative targets that might be regulated at the post-transcriptional or translational levels. The rules used for the target predictions were the same as previously reported [36,37]. To investigate the putative functions of the potential target genes, the target sequences were annotated using diverse protein databases [38], including Gene Ontology (GO), Cluster of Orthologous Groups (COG) and Kyoto Encyclopedia of Genes and Genomes (KEGG).

### Differential expression of miRNAs

IDEG6 software was used to select differentially expressed miRNAs among the six libraries, the chi-squared test was used to determine the statistical significance of the differences between the different libraries. When the false discovery rate was below 0.01, and the highest copy number was 2-fold higher than the lowest copy number, the miRNA was considered to be significantly different between different samples.

### Quantitative real-time PCR (qRT-PCR)

qRT-PCR was used to validate the sequencing results. Total RNA was extracted from the leaves using Trizol, digested with DNase I to eliminate genomic DNA contamination. cDNA was synthesized from total RNA using the miRcute miRNA First-Strand cDNA Synthesis kit (Tiangen, Beijing, China), according to the manufacturer’s instructions. All qRT-PCR reactions were performed in 96-well plates on a Chromo4 Real-Time PCR System (Bio-Rad, Hercules, USA) using SYBR Green PCR Master Mix Reagent (Tiangen, Beijing, China) with Opticon Monitor detection software. The reactions were performed in a volume of 20 μL containing 2 μL cDNA, 10 μL SuperReal PreMix (Tiangen), and 250 nM each of the universal reverse primers and the specific forward primers. The following reaction conditions were used: an initial polymerase activation step for 15 min at 94°C, followed by 40 cycles of denaturation for 10 s at 94°C, annealing for 20 s at 60°C, and elongation for 30 s at 72°C. A melting curve analysis was performed over a temperature range of 60–95°C, with stepwise 1°C increments in the temperature to verify specific amplification. Each sample was processed in triplicate, and 5.8 S rRNA was used as an internal control. To analyze the targets expression through q-PCR, the cDNA was synthesized from 2.0 μg total RNA using M-MLV Reverse Transcriptase (Tiangen, Beijing, China) and oligo-d (T) according to the manufacturer’s instructions. Similar reaction conditions were performed to detect the miRNAs, except the templates were replaced with cDNA for the targets and the primers were replaced with gene-specific primers. The relative abundances were normalized to an internal actin control [39] and analyzed according to a previously reported method [40].

### Data analysis

The raw sequence data are available at the DDBJ Sequence Read Archive under Submission of soybean_lab-0002 in DNA databank of Japan, accession number DRA003269.

## Results

### Sequencing of small RNAs from soybean

**Fig 1:**
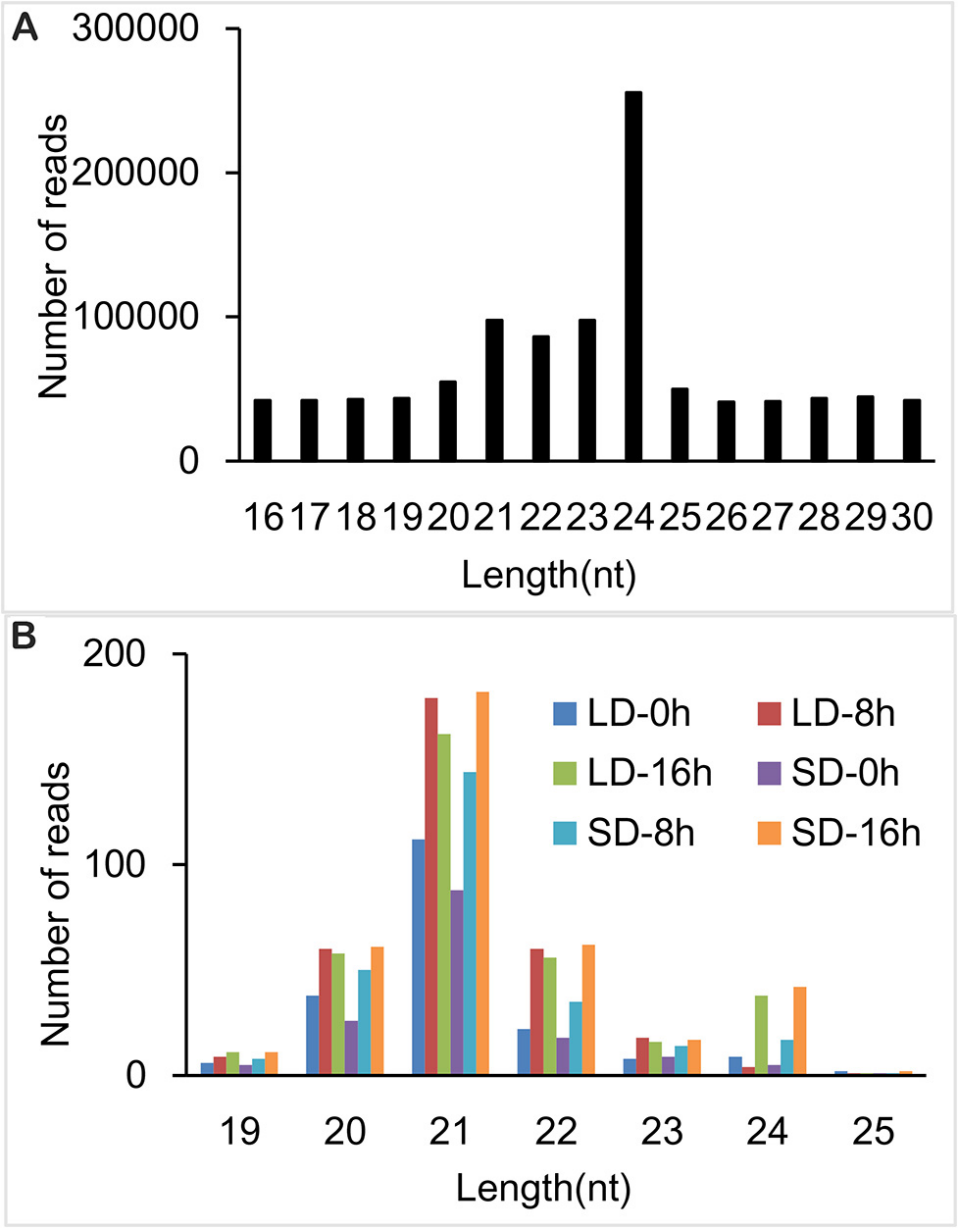
Size distribution of the clean tags after preliminary analysis of the sequencing mapped to the database named miRBase. (A) The size distribution of the clean tags. The clean tags could be mapped to the soybean genome, but were not observed among known noncoding RNAs through BLAST against the Rfame (a database of noncoding RNAs). These results were compared with the miRBase (a database of known miRNAs). (B) The size distribution of the tags of the six libraries mapped to the miRBase.

The next-generation sequencing using Illumina Cluster Station and Illumina Genome Analyzer provided a large number of reads from both the SD- and LD-treated RNA pools (pools A were from 0 h, pools B were from 8 h and pools C were from 16 h after treatment) of soybean cultivar ‘DongNong 42’. There were many non-protein coding genetic reads among the pools. After preliminary analyses, the number and percentage of non-protein coding generic reads among the six libraries were identified, and the unique, potential protein coding, genetic reads were obtained (S1 Table). The number of potentially genetic reads in the soybean pools for LD-0 h (1,235,097) and LD-8 h (1,650,183) were less than the number of the tags in the soybean pool for LD-16 h (3,116,563). A similar trend was observed in pools for SD-0 (851,631), SD-8 (2,684,356) and SD-16 h (2,417,518). In total, 11,955,348 reads were obtained, ranging from 16 to 30 nucleotides (nt). Sequences with 21, 22, 23 and 24 nucleotides had the highest abundance (Fig 1A).

The unique reads of 76.74% to 91.13% could be mapped to the soybean ‘Williams 82’ genome using SOAP (S2 Table). The mapped reads were subsequently mapped to the Sanger Non-Coding (but genetic) RNA database (S3 Table). The majority of non-protein coding, genetic RNAs were derived from rRNAs and were not included for the identification of the known and novel predicted miRNAs.

### Identification of known and novel predicted miRNAs related to light change

The miRBase is the primary online repository for all microRNA sequences and annotation. The current release (miRBase 21) contains over 35,828 microRNA gene loci from 223 species and 28,645 distinct mature microRNA sequences. To identify known soybean miRNAs in the six libraries, the clean reads were compared with known miRNA precursors and mature miRNA sequences in miRBase 21, allowing no more than two mismatches [29] (S4 Table). The results showed that sequences with 21, 22, 23 and 24 nucleotides had the highest abundance (Fig 1B). A total of 315 known miRNAs were identified in the six small RNA libraries. These miRNAs were classified into two groups. Group I comprised 162 non-conserved miRNAs exclusively present in *G*. *max*. Group II contained 155 miRNAs highly conserved across many of the 206 species. These molecules belonged to 28 miRNA families. The largest conserved family was miR156 with 15 members (S5 Table).

In total, 81 novel miRNA candidates were identified among the six libraries. Most of the novel predicted miRNAs had relatively low abundances and were exclusively observed in one or two libraries. However, miR-60-3p was observed in five libraries (but not in LD-0 h). None of novel predicted miRNAs was detected in the all six libraries (S8 Table).

### Prediction of miRNA targets

In plants, mismatches around the center of miRNA/mRNA complementary regions typically reduce gene expression during translation [41]. These mismatches can be detected using psRNATarget software, and the results suggest potential translational inhibition [42]. Therefore, PsRNATarget software was used to predict the targets of the conserved and novel predicted miRNA obtained from the present study.

A total of 255 unique targets for 65 known miRNA families and 154 unique targets for 27 novel miRNA families were obtained. Further analyses indicated that some miRNAs targeted a single gene (*i*.*e*., miR168 and miR2109), while the other miRNAs targeted multiple genes (*i*.*e*., miR156 and miR172).

**Fig 2:**
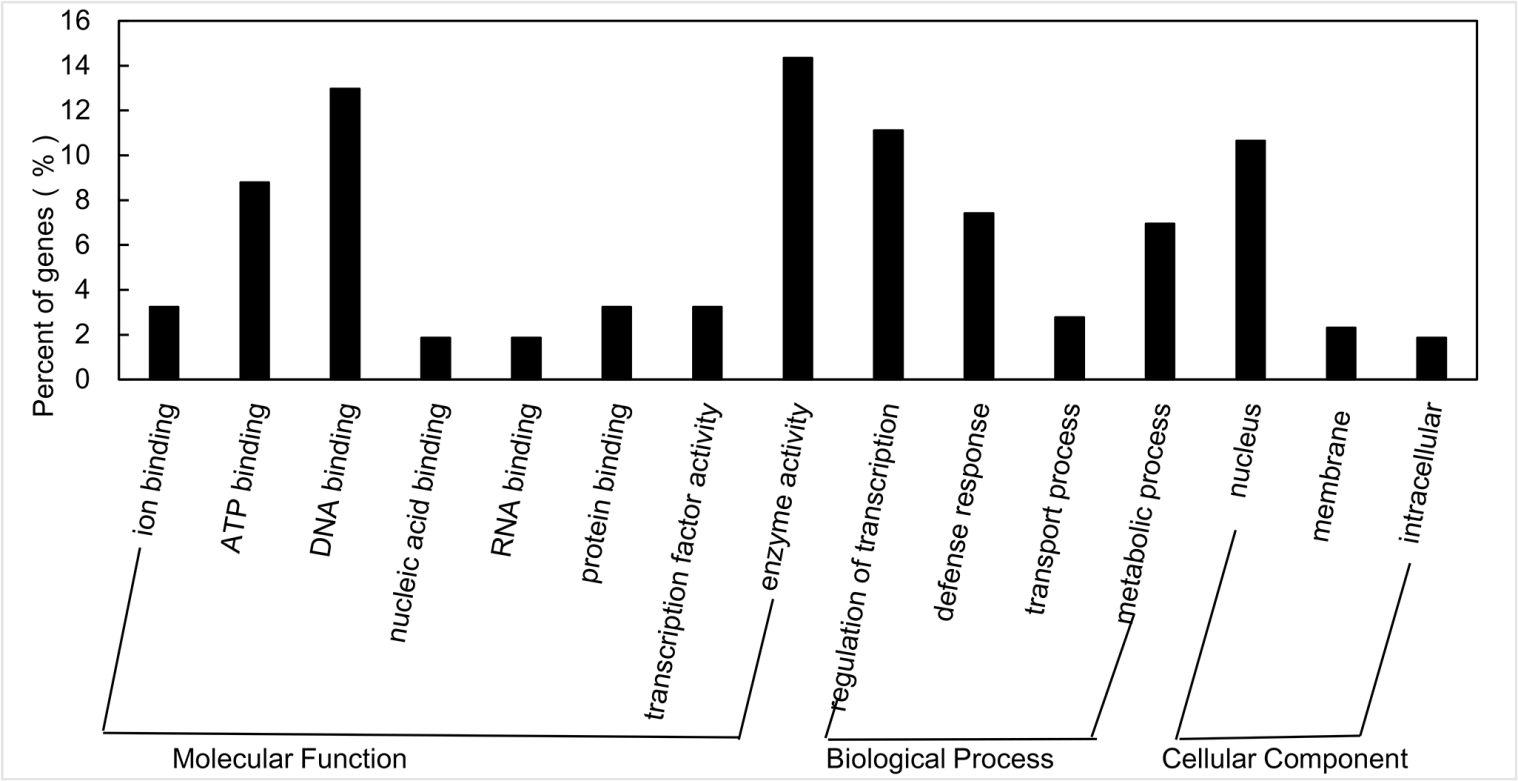
GO categories and distribution of miRNA targets in soybean. GO analysis was used to evaluate the potential functions of these miRNA targets.

The potential functions of the miRNAs targets were predicated using GO analysis [38,43]. Among them 32 genes were implicated in cellular components, while 61 genes were implicated in biological processes and 123 genes in molecular functions. Among the genes involved in the biological processes, the most over-represented GO terms were DNA binding, protein binding, ATP binding and the regulatory transcription factors (Fig 2).

**Fig 3:**
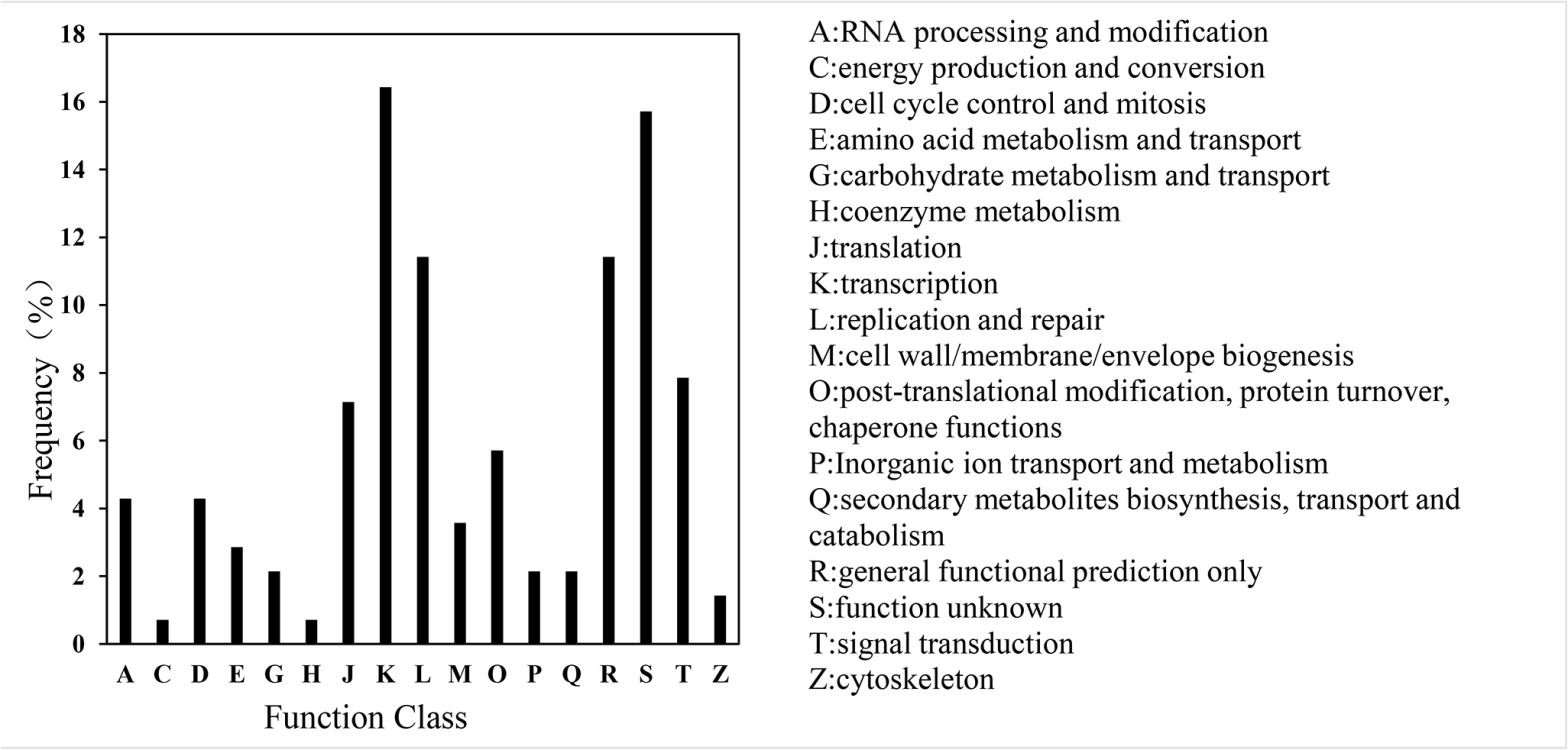
COG functional classification of consensus sequences in soybean. COG analysis was used to further evaluate the completeness of the transcriptome and the effectiveness of the annotation process.

The Clusters of Orthologous Genes (COG) analysis was used to further understand the function of the targets [38,44]. More than 140 sequences were assigned to COGs of at least seventeen functional classes. The largest group was transcription, followed by function unknown, general functional prediction, replication and repair, signal transduction and translation (Fig 3). To further examine the biological interpretation of the targets, the KEGG analysis was used [38]. Among the twenty-seven different pathways observed, the most frequently represented pathways were signal transduction and DNA replication and repair; fourteen targets functioned as transcription factors in signal transduction pathways, and six targets functioned in replication and repair.

### Differential abundances of miRNAs associated with day-length changes

The differential abundance of both known and novel predicted microRNAs was analyzed using IDEG6 software. The induction ratio was calculated as an increase in the relative abundance under LD conditions compared with the abundance under SD conditions. This molecule was only considered as a light-deficiency responsive miRNA when the induction ratios were significantly changed between different libraries.

**Fig 4:**
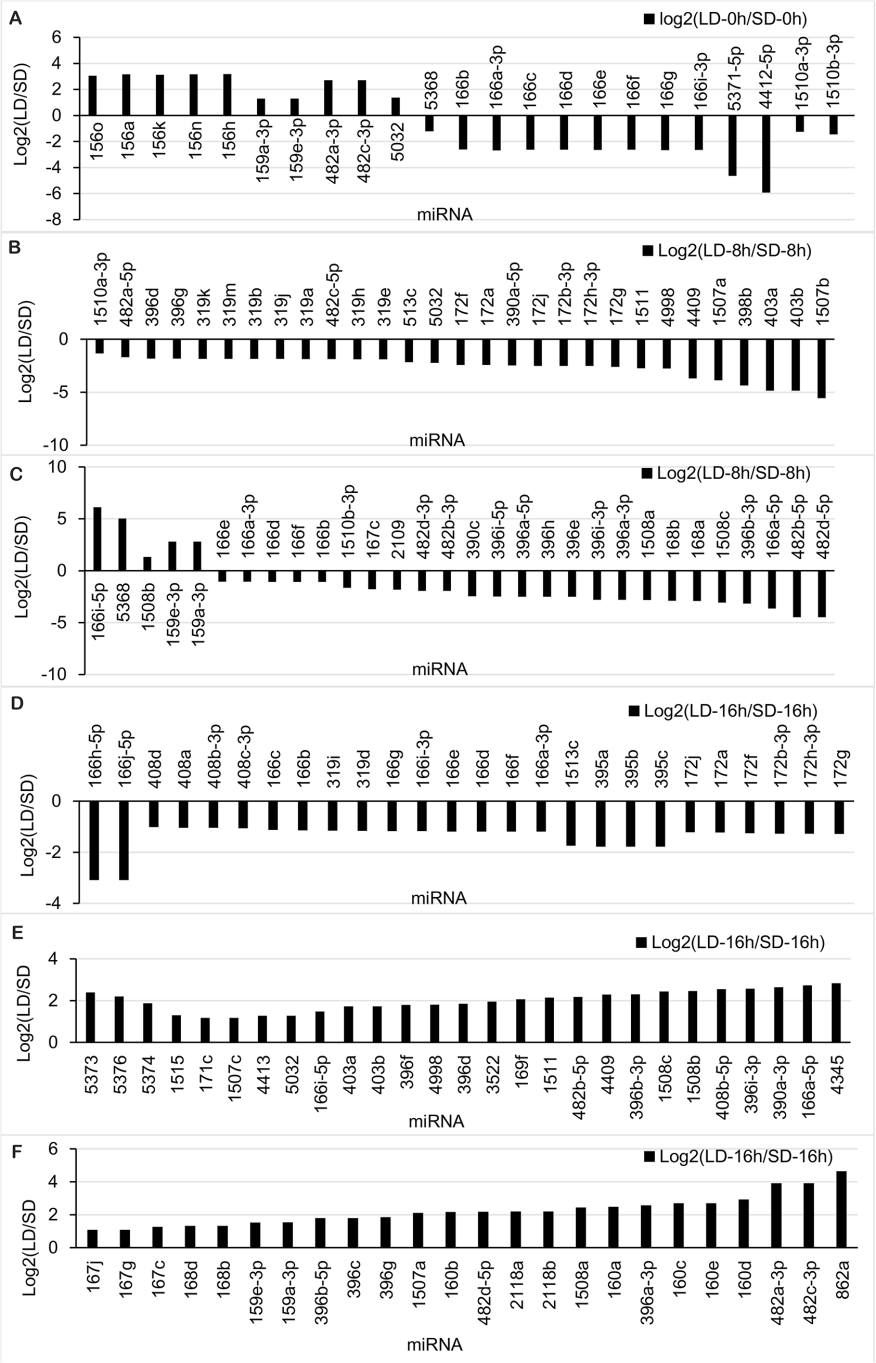
Differential expression analysis of soybean miRNAs identified using Solexa sequencing. The software IDE6 was used to analyze the known and novel predicted miRNAs obtained through high-throughput sequencing. Panels **a** to **f** show LD to SD abundance differences for time points A to C.

There were 23, 59 and 77 known miRNAs showing different abundance profiles at the time points 0, 8 and 16 h, respectively (Fig 4). At 0 h, 13 known miRNAs were decreased, while another 10 known miRNAs increased under LD treatments. At 8 h, only five known miRNAs were up-regulated, while another 54 known miRNAs were down-regulated under LD treatment. At 16 h, 26 known miRNAs were down-regulated, while another 51 known miRNAs were up-regulated under LD treatment.

Among the *G*. *max*-specific miRNAs, miR1510a-3p was the most abundant (459, 4,011,4074, 192, 1,587 and 2,416 reads in the six libraries, 0–16 h SDs and LDs), followed by miR1510b-3p and miR4412 (S5 Table). Most of *G*. *max*-specific miRNAs had relatively lower abundances. Among the conserved miRNAs, the most abundant reads in the six libraries encoded miR159a-3p (with 698, 12,935, 6,559, 284, 1,854, and 19,034 reads) and miR159e-3p (with 702, 12,962, 6,611, 286, 1,864, and 19,093 reads) (S5 Table). The data showed that the conserved miRNAs had higher abundances compared with the non-conserved and species-specific miRNAs, inferring that the conserved miRNAs have more important functions in plants [45].

Among the 81 novel predicted miRNAs of soybean, miR35-5p was the most abundant, with 314 reads under LDs at 8 h, and 237 reads under SDs at 8 h. The presence of miR60-3p was detected in five libraries, except under LDs at 0 h. While no miRNAs were consistent among all libraries (S8 Table).

### Experimental validation of the miRNAs and their targets

**Fig 5:**
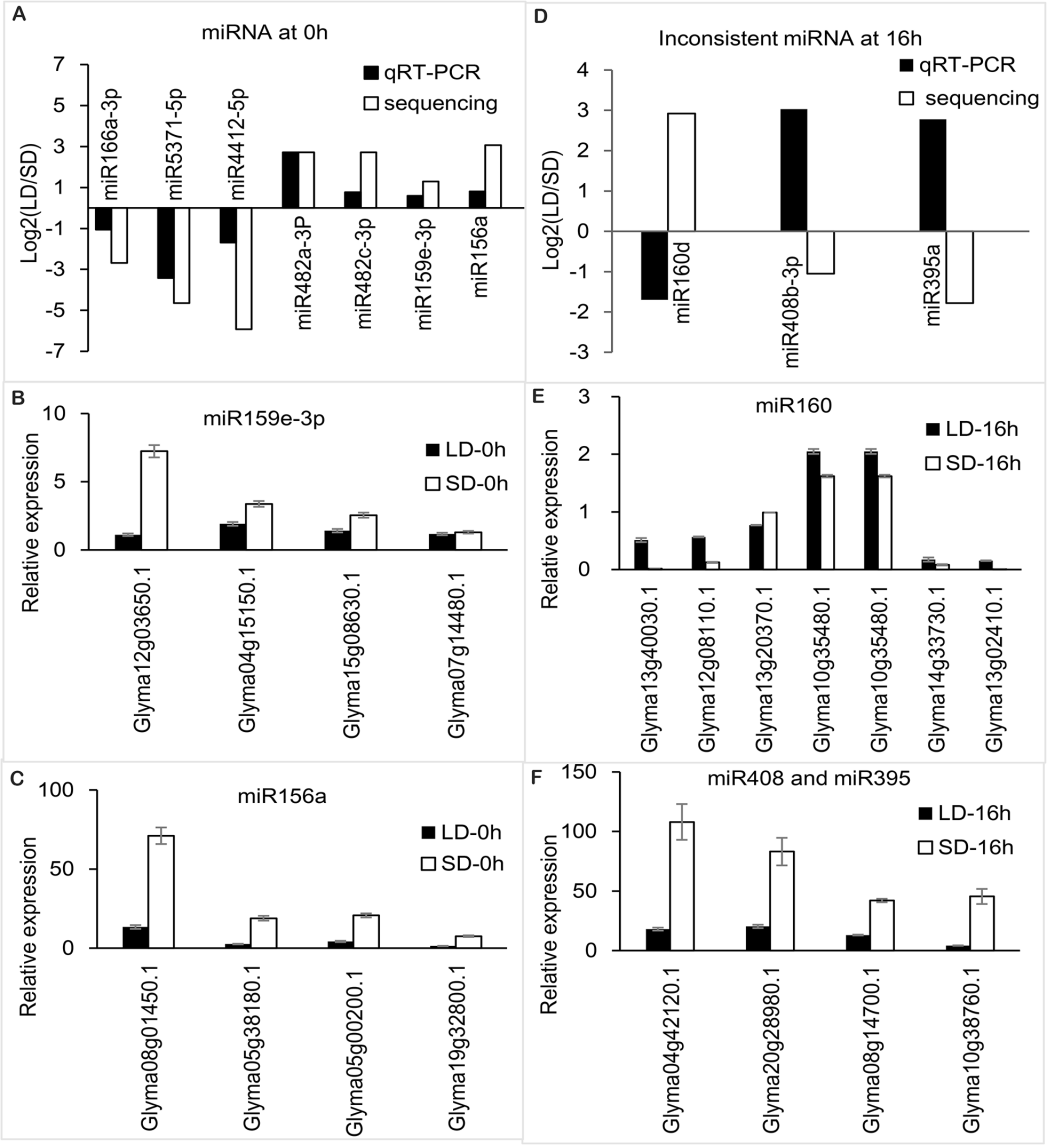
qRT-PCR validation of some miRNAs and their targets identified by Solexa sequencing. qRT-PCR reactions were used to verify the sequencing and the target predictions. (A) The result of the qRT-PCR validation of some miRNAs were identified through sequencing. The expression was represented as the ratios of the expression under SD treatment to that under LD treatment, and the 5SrRNA was used as a control. (B) The results of the qRT-PCR validation of the target predictions for miR159e-3p. The expression was presented as the ratio of expression under SD and LD treatment, and the 18SrRNA acted as a beta-actin (C) The result of the qRT-PCR validation of the target predictions for miR156a. (D) The result of qRT-PCR validation of the target predictions for miR160. (E) The results of qRT-PCR validation of the target predictions for miR395 and miR408. (F) The result of qRT-PCR validation of some miRNAs identified by sequencing.

Stem-loop qRT-PCR was used to verify the day length-responsive miRNAs obtained through differential abundance analyses. Seven miRNAs at 0 h, 23 miRNAs (including four novel predicted miRNAs) at 8 h and 19 miRNAs (including one novel predicted miRNA) at 16 h were selected to verify the sequencing results (Fig 5A, S1 File and S9 Table). The data indicated that the abundance of most microRNAs selected were consistent with the sequencing results, except for three miRNAs at 16 h (miR160, miR408b-3p and miR395a) (Fig 5D). The induction ratio of most of the microRNAs selected was higher for the miRNA sequencing analysis than in the qRT-PCR validation experiment, except for four miRNAs (miR4998, miR4413b, miR396 and miR1511) at 16 h (S1 File).

The low abundance of novel predicted and species-specific miRNAs using stem-loop qRT-PCR made these molecules difficult to detect. Therefore, only a few novel predicted miRNAs (soybean-miR-36, soybean-miR-64, soybean-miR-35, soybean-miR-57 and soybean-miR-60), showing greater abundance than others, were selected to design primers for qRT-PCR validation (Table 1 and S8 Table). The results of the qRT-PCR showed that the expression of soybean-miR-36 at 8 h and soybean-miR-64 at 16 h were increased under SDs (Fig C and Fig E in S1 File). The expression of soybean-miR-35, soybean-miR-57 and soybean-miR-60 at 8 h were decreased under SDs [Fig C in S1 File]. Neither soybean-miR-4 at 0 h nor soybean-miR-77 at 16 h could be detected.

**Table 1:** The novel soybean miRNAs verified using qRT-PCR.

miRNA ID	Sequence (5’-3’)	Length (nt)	Start/end precursor	Length of precursors (nt)	Arm	MFE (kcal/mol)
**Soybean-miR-4**	AAUGAGAACUUUGAAGGCCGAA	22	9955786–9955874	88	3p	-18.10
**Soybean-miR-35**	GGAAUGGGCUGAUUGGGAA	19	7783821–7783912	92	5p	-45.70
**Soybean-miR-36**	GGAGUGAAAUAGAACAUGAAA	21	13036509–13036600	91	5p	-20.00
**Soybean-miR-57**	UAUGGGGGGAUUGGGAAGGAA	21	35360317–35360403	86	5p	-40.30
**Soybean-miR-60**	UCUUCCCUACACCUCCCAUACC	22	35360314–35360403	89	3p	-43.50
**Soybean-miR-64**	UGAGAACUUUGAAGGCCGAA	20	9955786–9955874	88	3p	-18.10
**Soybean-miR-77**	UUCUGAACCAGACUUUGAUGUC	22	20678566–20678664	98	5p	-25.50

The primers for 65 targets belonging to 13 miRNAs were designed to verify the prediction of miRNAs targets (S10 Table). The results indicated that the expression patterns of the targets showed a negative relationship with the corresponding miRNAs (Fig 5, S1 and S2 Files). Four targets of miR159 at 0 h were increased under SDs as the positive regulator of flowering time in soybean (Fig 5B). Among these, the most sensitive gene to day length change was *Glyma12g03650*.*1*, whose transcript abundance decreased six-fold under LDs compared with SDs, while *Glyma05g38180*.*1* and *Glyma07g14480*.*1* decreased 2- and 1.1-fold, respectively.

Four targets of miR156 at 0 h were increased under SDs, functioning as the positive regulators of the flowering time in soybean (Fig 5C). The transcript abundance of *Glyma19g32800*.*1* (*SPL9*), *Glyma05g38180*.*1* (*SPL13*), *Glyma08g01450*.*1* (*SPL13*) and *Glyma05g00200*.*1* (*SPL12*) were significantly decreased five to six-fold l under LDs compared with SDs.

At 8 h, the expression of the targets of miR1507, miR1508, miR482, miR1510 and miR166 were increased under LDs, indicating that these miRNAs responded to day-length changes (Fig A-D in S2 File), and the miRNAs were inferred as negative regulators of flowering time in soybean. Two gene (*Glyma04g29220*.*1* and *Glyma05g09220*.*1*) transcripts encoding targets of miR1507 responded to light treatment (Fig B in S2 File), and the expression of these transcript increased six- and five-fold under LDs compared with SDs, respectively.

The transcript abundance of two targets of miR1508 (*Glyma09g30500*.*1* and *Glyma16g28020*.*1*) increased four- and nine-fold under LDs, respectively, compared with SDs (Fig B in S2 File). The transcript abundance of *Glyma07g33600*.*1*, the target of miR482, increased four-fold under LDs compared with SDs (Fig A in S2 File). Three targets of miR1510 (*Glyma04g39740*.*1*, *Glyma16g27540*.*1* and *Glyma19g07660*.*1*) responded to day-length changes, with three- to four-fold increases under LDs compared with SDs (Fig D in S2 File).

The expression of four targets of miR166 at 8 h was verified through qRT-PCR (Fig C in S2 File). Only *Glyma04g09000*.*1* responded to light treatment, showing a three-fold increase under LDs compared with SDs. Four miRNA159 target genes (*Glyma07g14480*.*1*, *Glyma12g03650*.*1*, *Glyma04g15150*.*1* and *Glyma07g33600*.*1*) were increased under SDs, showing a two- to four-fold increase under LDs (Fig A in S2 File). Therefore, these genes were inferred as positive regulators of flowering time in soybean.

At 16 h, the targets of miR159, miR1508, miR396, and miR4413 showed increased transcription under SDs and responded to the day-length treatment. These miRNAs were inferred as positive regulators of the flowering time in soybean (Fig E-G in S2 File). Among the three target genes of miR159, *Glyma07g14480*.*1* and *Glyma12g03650*.*1* responded strongly to light treatment, showing a 28- and 35-fold increase under SDs, respectively, while the transcript abundance of *Glyma15g08630*.*1* increased three-fold under SDs (Fig F in S2 File).

The two target genes of miR1508 (*Glyma09g30500*.*1* and *Glyma16g28020*.*1*) and one target gene of miR396 (*Glyma16g00260*.*1)* responded strongly to light treatment, showing a two- to eight-fold increase under SDs (Fig F-G in S File). Six of the nine target genes of miR4413 responded to light treatment and increased two-fold (*Glyma09g30940*.*1*), three-fold *(Glyma09g07300*.*1* and *Glyma14g38270*.*1*), four-fold (*Glyma07g11290*.*1* and *Glyma07g11410*.*1*) and eight-fold (*Glyma16g25410*.*1*) under SDs, respectively (Fig E in S2 File).

## Discussion

MiRNAs play important roles at different development stages of plant growth through the regulation of the expression of targets at transcriptional and post-transcriptional levels [10, 19, 30, 33, 46]. Since the first miRNA was identified in 1993 [14], 28,645 hairpin sequences and 35,828 mature sequences have been recorded in the miRBase [16,17]. As a new technology, high throughput sequencing methods are frequently used in the identification of miRNAs in plants [19,28–30, 33, 47–52]. Until recently, 1215 miRNAs of *G*. *max* were reported in the miRBase. Among these, a number of soybean miRNAs were identified using high-throughput sequencing, combined with experimental validation (Northern hybridization or qRT-PCR), including root-related miRNAs [32], abiotic and biotic stress-related miRNAs [28], aluminum stress-related miRNAs [29] and seed development-related miRNAs [30]. As a SD plant, the day length affects the output of soybean through the regulation of the direct transmission from vegetative to reproduction growth. However, day length-related miRNAs have not yet been reported.

Here, we used high-throughput sequencing and stem-loop qRT-PCR analysis to identify the miRNAs of soybean in response to day length changes. The accuracy of the sequencing was verified through qRT-PCR, and the expression pattern of the miRNAs and the miRNA targets in response to day length change were also analyzed.

### The accuracy of high-throughput sequencing

The clean tags were mapped to the soybean genome to verify the accuracy of the high-throughput sequencing. The data showed that 79.61%, 91.13%, 87.19%, 76.74%, 88.78% and 90.71% of the tags in the LD-treated libraries (from time points 8 h to 16 h) and SD treated libraries (from time points 0 h to 16 h) were mapped to the soybean genome, indicating that the accuracy of the high-throughput sequencing was high.

The size distribution of the clean tags and the known microRNAs was obtained to verify the accuracy of the high-throughput sequencing. The clean tags were used to obtain the size distribution of the tags, and the results indicated that sequences with 21, 22, 23 and 24 nucleotides had higher abundance. The known microRNAs, which could be mapped to the miRBase, were used to obtain the size distribution of the known microRNAs. The results showed that the sequences with 21 nucleotides had the highest abundance. The size distribution of the clean tags, and the known microRNAs were consistent with previous studies [28, 30, 32, 48, 49], suggesting that the accuracy of the high-throughput sequencing was high.

QRT-PCR was used to verify the miRNAs obtained through sequence analysis. In total, three miRNAs were increased and four miRNAs were decreased under SDs at 0 h. Three miRNAs (including one novel predicted miRNA) were decreased and 20 miRNAs (including three novel predicted miRNAs) were increased under SDs at 8 h. A total of 15 miRNAs (including one novel predicted miRNA) were decreased and one miRNAs was increased under SDs at 16 h. The expression of these miRNAs was consistent with the results obtained through the sequence analysis, except for three miRNAs at 16 h. The results indicated that the accuracy of the high-throughput sequencing was high.

### The expression pattern of miR156, miR159 and miR172

It has been reported that miR156 targets *SPL* genes and plays an important role in a separate endogenous pathway [53]. At the shoot apex, the forward feed action of miR156/*SPL* and *FT/FD* modules worked together for the regulation of flowering [53]. In Arabidopsis, the *SPLs* were divided into two groups. One group encoded small proteins primarily comprising the SBP DNA binding domain that primarily function in the control of flowering time and phase changes, such as *SPL3*, *SPL4* and *SPL5*, and the other group encoded much larger proteins that affect leaf initiation rates, such as *SPL9* and *SPL15* [53–55]. Previous studies have reported that the overexpression of miR156 delayed the onset of flowering, while the overexpression of *SPL3* and *SPL9* accelerated flowering, indicating that both groups of *SPL* factors were important for flowering [53, 56–58].

Here, differentially abundant miRNA expression under different day-length treatments revealed that the induction of five members of the miR156 family (miR156a, miR156h, miR156k, miR156n and miR156o) was significantly increased under LDs. Four differentially expressed targets of miRNA156, belonging to the *SPL* family, were further verified through qRT-PCR. The transcript abundance of *Glyma08g01450*.*1 (SPL13)*, *Glyma19g32800*.*1 (SPL9)*, *Glyma05g00200*.*1 (SPL12)* and *Glyma05g38180*.*1 (SPL13)* were five-, six-, five- and six-fold decreased under LDs compared with SDs, respectively. These results above indicated that the increase of miR156 abundance in LDs resulted in a decline in *SPLs* in soybean. The reduction in SPL activities through increased miR156 expression delayed the onset of flowering in SD plants, such as soybean, similar to the LD plant Arabidopsis [57].

The gibberellin pathway has been shown to be the most important pathway for floral induction under short days [22,59]. *AtMYB33* expression was increased at the apex during floral initiation and proposed to interact with the *LFY* promoter motif to promote flowering [60,61]. In cereals and Arabidopsis, *GAMYB* genes were predominantly expressed in the anthers and seeds, where less miR159 accumulation was observed [26]. This negative correlation in expression patterns provided evidence for miR159-directed *GAMYB* regulation. Plants overexpressing miR159a also showed decreased *MYB33* expression and were male sterile, with delayed flowering time [26], indicating that miR159 functions as a negative regulator of the flowering time through the GA signal pathway.

In the present study, the expression of miR159a-3p and miR159e-3p was significantly decreased under SDs at 0, 8 and 16 h. In total, four differentially expressed targets of miR159, belonging to the *R2R3-MYB* family, were confirmed to respond to day length change through qRT-PCR. The abundance of *Glyma04g15150*.*1*, *Glyma12g03650*.*1*, *Glyma05g38180*.*1* and *Glyma07g14480*.*1* was two- to six-fold decreased under SDs compared with LDs at 0, 8 and 16 h. These results indicated that day length-dependent decreases in miR159 abundance in LDs subsequently decreased *R2R3-MYBs* in soybean, and the decrease in *R2R3-MYB* activity through miR159 promoted the onset of flowering in soybean under SDs. These results suggested that miR159 and its target genes are not only involved in the GA signal pathway but also in the photoperiod pathway. Further study is needed to determine the function of miR159 and its targets in the response to the day length changes.

MiR172, together with its six target genes (*AP2*, *TOE1*, *TOE2*, *TOE3*, *SMZ and SNZ*), functions in the regulation of flowering time. These molecules depend on *GI* but do not require a functional *CO* in Arabidopsis [23]. The overexpression of miR172 accelerated flowering. However, the argument concerning whether miR172 is regulated through day length remains [22,62]. In the present study, the expression of the six members of miR172 (a, b-3p, f, g, h-3p and j) were increased under SDs at 0 and 8 h, likely acting as a positive regulator of flowering in soybean. Among the 16 genes predicted as the targets of miR172, ten genes belonging to the *AP2-like* family were verified. Therefore, miR172, together with its targets, could affect flowering time and are likely regulated through day length in soybean.

### Other differentially expressed miRNAs

Currently, the other differentially expressed miRNAs regulated through day length in soybean have not been implicated in the regulation of flowering time in other plants. Few reports of species-specific, non-conserved microRNAs of soybean exist, except for miR5371 and miR5368 (involved in abiotic and biotic stresses) [28] and miR4412 and miR4998 (involved in the shoot apical meristem of soybean) [31]. In addition, the results of the present study revealed conserved miRNAs detected in other plants, such as miR166 (regulating shoot apical meristem and floral development in Arabidopsis) [63, 64], miR482 (in resistance to disease or abiotic stress *via NBS-LRR* proteins) [65], miR319 (affecting organ development and the processes of phase change in Arabidopsis) [46], miR160 (responses to the plant hormone auxin) [66], miR167 (involved in the floral organ formation) [27], miR390 (influence not only vegetative developmental transitions but also organ polarity in flowering plants) [67, 68], miR395 (regulating sulfate accumulation and allocation) [69], and miR408 (influenced through a variety of environmental conditions) [70, 71]. These molecules have not been previously reported, but confirmed to respond to the day length changes in the present study.

## Conclusions

Here, a number of conserved and un-conserved miRNA candidates from soybean were obtained through high-throughput sequencing. Sequence analysis was used to identify miRNAs with different reads among the six libraries. The accuracy of the sequencing and further bioinformatics analyses were verified through qRT-PCR. Seven miRNAs at 0 h, 23 miRNAs (four novel predicted miRNAs involved) at 8 h and 16 miRNAs (including one novel predicted miRNA) at 16 h responding to the day length changes were identified. However, further studies of these miRNAs and their target genes are needed to better understand the network for regulating flowering time in soybean.

## Supporting Information

S1 File qRT-PCR validation of soybean miRNAs identified through Solexa sequencing at 8 and 16 h. **qRT-PCR were used to verify the sequencing.** (Fig **A)** The results of the qRT-PCR validation of the conserved miRNAs identified through sequencing at 8 h. The expression was represented as the ratio of the expression under SD treatment to that under LD treatment, and the 5SrRNA was used as a control. (Fig **B)** The results of the qRT-PCR validation of non-conserved miRNAs identified through sequencing at 8 h. (Fig **C)** The results of the qRT-PCR validation of novel predicted miRNAs identified through sequencing at 8 h. (Fig **D)** The results of the qRT-PCR validation of conserved miRNAs identified through sequencing at 16 h. (Fig **E)** The results of the qRT-PCR validation of novel and non-conserved miRNAs identified through sequencing at 16 h.
(TIF)

S2 File qRT-PCR validation of the targets at 8 and 16 h. qRT-PCR was used to verify the targets prediction at 8 and 16 h. (Fig **A)** The results of qRT-PCR validation of the targets of miR159e-3p and miR482b-3p at 8 h. (Fig **B)** The results of the qRT-PCR validation of the targets of miR1507 and miR1508 at 8 h. (Fig **C)** The results of the qRT-PCR validation of the targets of miR166 at 8 h. (Fig **D)** The results of the qRT-PCR validation of the targets of miR1510 at 8 h. (Fig **E)** The results of the qRT-PCR validation of the targets of miR4413 at 16 h. (Fig **F)** The results of the qRT-PCR validation of the targets of miR396 and miR159e-3p at 16 h. (Fig **G)** The results of the qRT-PCR validation of the targets of miR1508 and miR4413 (the expression of the two targets were much higher than other targets of miR4413). at 16 h.
(TIF)

S1 Table Tags statistics of the preliminary analysis of the sequencing. There were many non-protein coding genetic reads among the six pools. After the preliminary analysis, the clean and unique reads were obtained.
(DOCX)

S2 Table Tags statistics of soybean genome alignment. The unique reads were mapped to the *Glycine max* ‘Williams 82’ genome using the SOAP program. The number and percentage of reads mapped are shown in S2 Table.
(DOCX)

S3 Table Tags statistics from the Rfam alignment. The mapped reads were mapped to the Sanger Non-Coding (but genetic) RNA database to note the Non-Coding RNA. The number and percentage of the Non-Coding RNA are shown in S3 Table.
(DOCX)

S4 Table Tags statistics from the miRBase alignment. The clean reads were compared with known miRNA precursors or mature miRNAs in the miRBase. The results of the six pools are shown.
(DOCX)

S5 Table The reads of the known miRNAs at the six libraries. The reads of the known miRNAs in the six libraries were obtained for further differential expression analyses.
(XLSX)

S6 Table Tags statistics of the novel miRNA. The novel predicted miRNAs were predicted through MIREAP.
(DOCX)

S7 Table The function analysis of the target-genes and its locus that was targeted using miRNAs. GO and KEGG analyses were used for the function analysis of the target genes.
(XLSX)

S8 Table The reads of the novel predicted miRNAs at the six libraries. (XLSX)

S9 Table The primer sequence of the miRNAs verified using qRT-PCR. (DOCX)

S10 Table The primer sequence of the target-genes verified using qRT-PCR. (DOCX)
