# Supplementary figures and images for: Identification of MicroRNAs in Response to Different Day Lengths in Soybean Using High-Throughput Sequencing and qRT-PCR
Source: PLoS One. 2015 Jul 10;10(7):e0132621. doi: 10.1371/journal.pone.0132621 (PMC4498749; doi:10.1371/journal.pone.0132621)

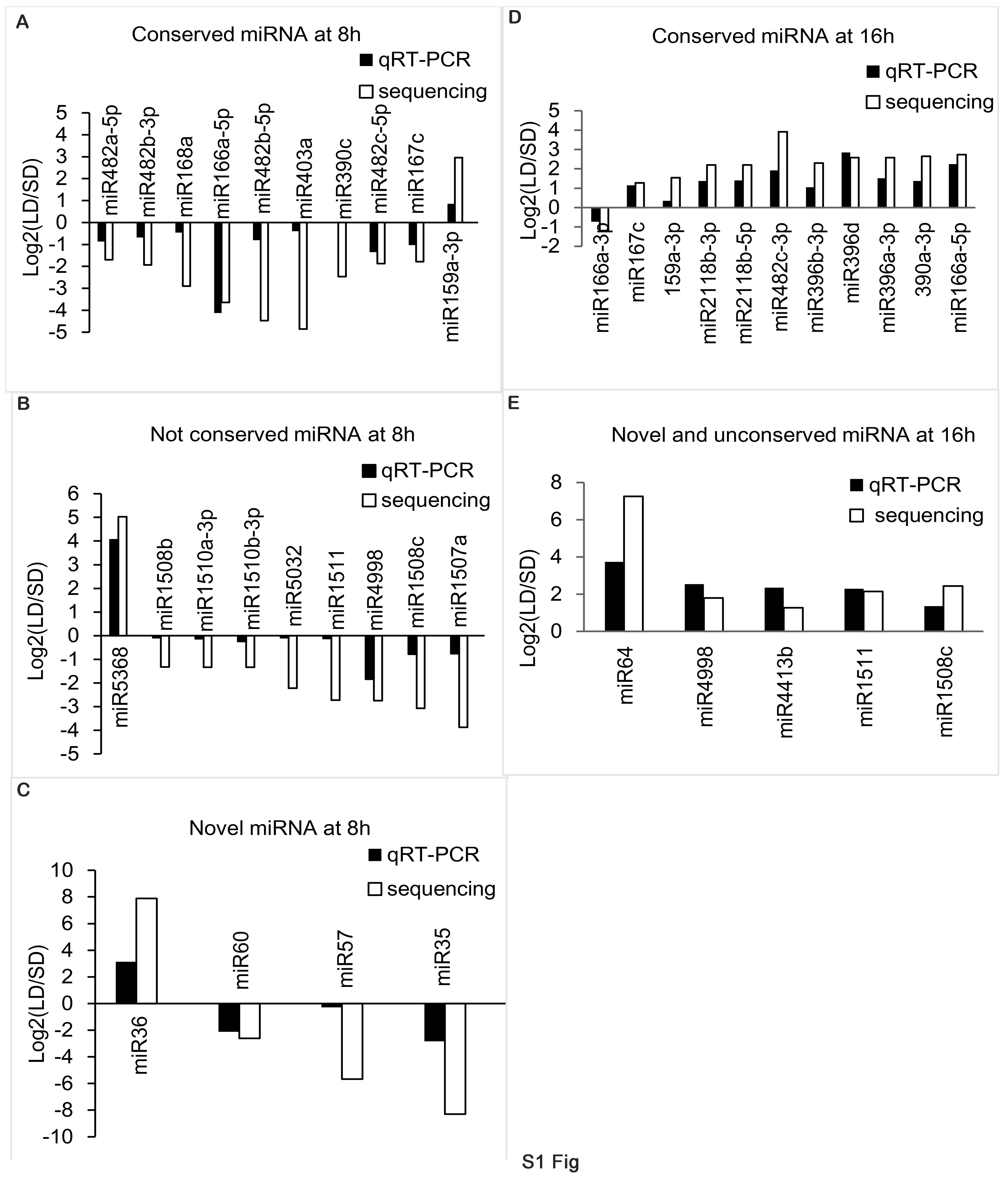

Supplement: S1 File — qRT-PCR were used to verify the sequencing. (Fig A) The results of the qRT-PCR validation of the conserved miRNAs identified through sequencing at 8 h. The expression was represented as the ratio of the expression under SD treatment to that under LD treatment, and the 5SrRNA was used as a control. (Fig B) The results of the qRT-PCR validation of non-conserved miRNAs identified through sequencing at 8 h. (Fig C) The results of the qRT-PCR validation of novel predicted miRNAs identified through sequencing at 8 h. (Fig D) The results of the qRT-PCR validation of conserved miRNAs identified through sequencing at 16 h. (Fig E) The results of the qRT-PCR validation of novel and non-conserved miRNAs identified through sequencing at 16 h. (TIF) [file pone.0132621.s001.tif]

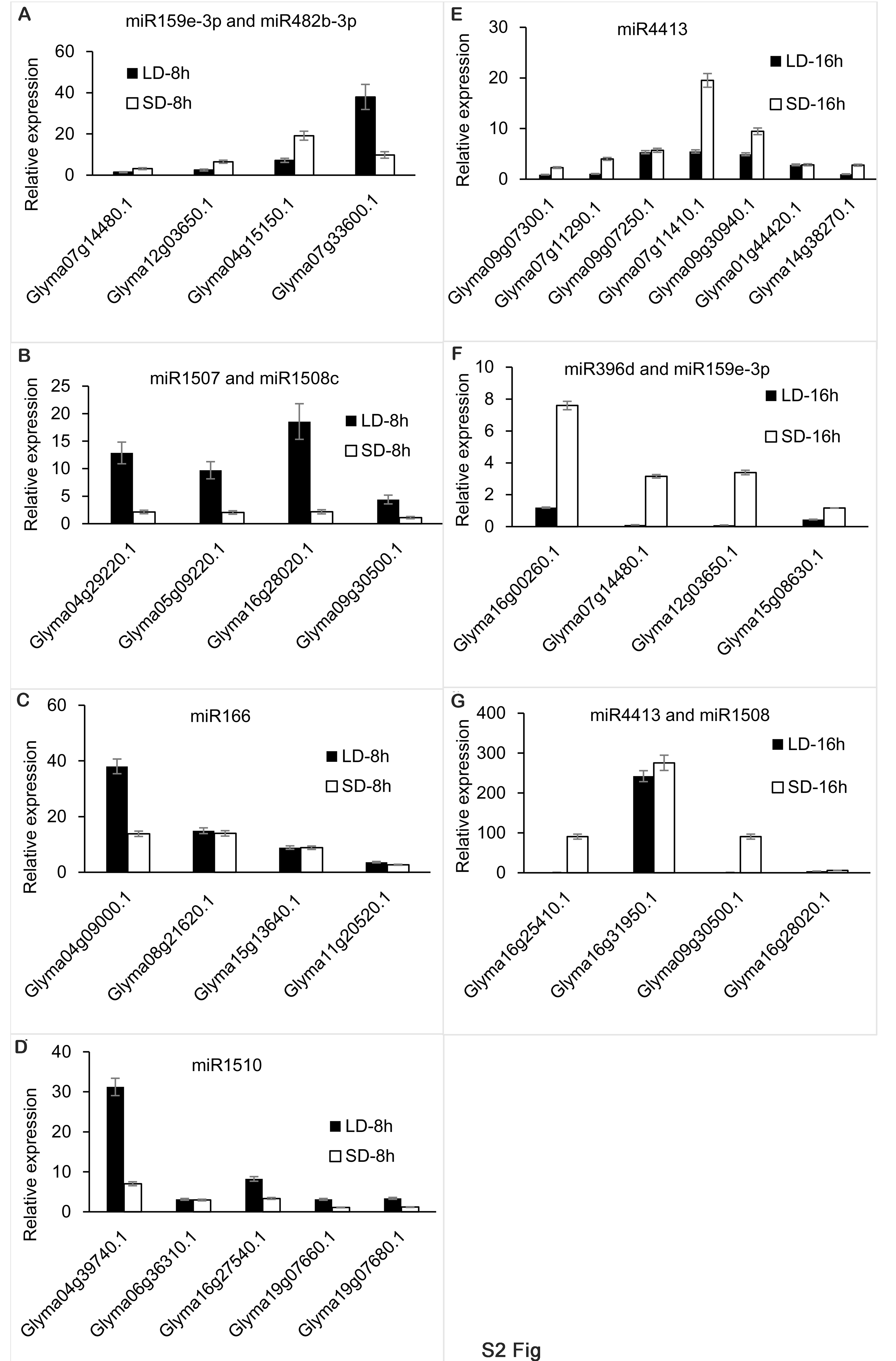

Supplement: S2 File — qRT-PCR was used to verify the targets prediction at 8 and 16 h. (Fig A) The results of qRT-PCR validation of the targets of miR159e-3p and miR482b-3p at 8 h. (Fig B) The results of the qRT-PCR validation of the targets of miR1507 and miR1508 at 8 h. (Fig C) The results of the qRT-PCR validation of the targets of miR166 at 8 h. (Fig D) The results of the qRT-PCR validation of the targets of miR1510 at 8 h. (Fig E) The results of the qRT-PCR validation of the targets of miR4413 at 16 h. (Fig F) The results of the qRT-PCR validation of the targets of miR396 and miR159e-3p at 16 h. (Fig G) The results of the qRT-PCR validation of the targets of miR1508 and miR4413 (the expression of the two targets were much higher than other targets of miR4413). at 16 h. (TIF) [file pone.0132621.s002.tif]
