# Supplementary material for: Identification of MicroRNAs in Response to Different Day Lengths in Soybean Using High-Throughput Sequencing and qRT-PCR
Source: PLoS One. 2015 Jul 10;10(7):e0132621. doi: 10.1371/journal.pone.0132621 (PMC4498749; doi:10.1371/journal.pone.0132621)
Supplement: S1 Table — There were many non-protein coding genetic reads among the six pools. After the preliminary analysis, the clean and unique reads were obtained. (DOCX) [file pone.0132621.s003.docx]

**S1 Table. Tags statistics of the preliminary analysis of the sequencing**

|  | **Soybean_LD-0h** | | **Soybean_LD-8h** | | **Soybean_LD-16h** | | **Soybean_SD-0h** | | **Soybean_SD-8h** | | **Soybean_SD-16h** | | |
| --- | --- | --- | --- | --- | --- | --- | --- | --- | --- | --- | --- | --- | --- |
|  | **Number** | **Percent** | **Number** | **Percent** | **Number** | **Percent** | **Number** | **Percent** | **Number** | **Percent** | **Number** | **Percent** |  |
| **Total Tags number** | 3665092 | 100.00% | 3620396 | 100.00% | 6696225 | 100.00% | 5873890 | 100.00% | 5855711 | 100.00% | 6125149 | 100.00% |  |
| **Filter low quality Tags** | 797263 | 21.75% | 787469 | 21.75% | 1430091 | 21.36% | 1256748 | 21.40% | 1196092 | 20.43% | 1380635 | 22.54% |  |
| **Adaptor 3 insert null** | 9 | 0.00% | 3 | 0.00% | 3 | 0.00% | 10 | 0.00% | 3 | 0.00% | 3 | 0.00% |  |
| **5' adaptor contaminants** | 0 | 0.00% | 2 | 0.00% | 0 | 0.00% | 2 | 0.00% | 0 | 0.00% | 3 | 0.00% |  |
| **Length<16** | 121035 | 3.30% | 292231 | 8.07% | 341682 | 5.10% | 18022 | 0.31% | 450400 | 7.69% | 425056 | 6.94% |  |
| **Length>30** | 1491783 | 40.70% | 818626 | 22.61% | 1738915 | 25.97% | 3729374 | 63.49% | 1434532 | 24.50% | 1806555 | 29.49% |  |
| **PloyA** | 19905 | 0.54% | 71882 | 1.99% | 68971 | 1.03% | 18103 | 0.31% | 90328 | 1.54% | 95379 | 1.56% |  |
| **Clean number** | 1235097 | 33.70% | 1650183 | 45.58% | 3116563 | 46.54% | 851631 | 14.50% | 2684356 | 45.84% | 2417518 | 39.47% |  |
| **Uniq number** | 68062 | 1.86% | 212371 | 5.87% | 234296 | 3.50% | 48097 | 0.82% | 212374 | 3.63% | 248594 | 4.06% |  |
