# Supplementary material for: Identification of MicroRNAs in Response to Different Day Lengths in Soybean Using High-Throughput Sequencing and qRT-PCR
Source: PLoS One. 2015 Jul 10;10(7):e0132621. doi: 10.1371/journal.pone.0132621 (PMC4498749; doi:10.1371/journal.pone.0132621)
Supplement: S2 Table — The unique reads were mapped to the Glycine max ‘Williams 82’ genome using the SOAP program. The number and percentage of reads mapped are shown in S2 Table. (DOCX) [file pone.0132621.s004.docx]

**S2 Table. Tags statistics of soybean genome alignment.**

|  | **Soybean_LD-0h** | | **Soybean_LD-8h** | | **Soybean_LD-16h** | | **Soybean_SD-0h** | | **Soybean_SD-8h** | | **Soybean_SD-16h** | |
| --- | --- | --- | --- | --- | --- | --- | --- | --- | --- | --- | --- | --- |
|  | **Tags** | **Percent** | **Tags** | **Percent** | **Tags** | **Percent** | **Tags** | **Percent** | **Tags** | **Percent** | **Tags** | **Percent** |
| **Total** | 68062 | 100.00% | 212371 | 100.00% | 234296 | 100.00% | 48097 | 100.00% | 212374 | 100.00% | 248594 | 100.00% |
| **Mapped** | 54183 | 79.61% | 193535 | 91.13% | 204272 | 87.19% | 36912 | 76.74% | 188549 | 88.78% | 225508 | 90.71% |
| **Pefect map** | 21932 | 40.48% | 118326 | 61.14% | 108870 | 53.30% | 16365 | 44.34% | 86258 | 45.75% | 144299 | 63.99% |
| **Unpefect map** | 32251 | 59.52% | 75209 | 38.86% | 95402 | 46.70% | 20547 | 55.66% | 102291 | 54.25% | 81209 | 36.01% |
| **unmapped** | 13879 | 20.39% | 18836 | 8.87% | 30024 | 12.81% | 11185 | 23.26% | 23825 | 11.22% | 23086 | 9.29% |
