# Supplementary material for: Identification of MicroRNAs in Response to Different Day Lengths in Soybean Using High-Throughput Sequencing and qRT-PCR
Source: PLoS One. 2015 Jul 10;10(7):e0132621. doi: 10.1371/journal.pone.0132621 (PMC4498749; doi:10.1371/journal.pone.0132621)
Supplement: S3 Table — The mapped reads were mapped to the Sanger Non-Coding (but genetic) RNA database to note the Non-Coding RNA. The number and percentage of the Non-Coding RNA are shown in S3 Table. (DOCX) [file pone.0132621.s005.docx]

**S3 Table. Tags statistics of Rfam alignment.**

|  | **SD-0h** | | | | **SD-8h** | | | | **SD-16h** | | | |
| --- | --- | --- | --- | --- | --- | --- | --- | --- | --- | --- | --- | --- |
| **RNA_Types** | **Uniq_tags** | **Uniq_ratio** | **Total_tags** | **Total_ratio** | **Uniq_tags** | **Uniq_ratio** | **Total_tags** | **Total_ratio** | **Uniq_tags** | **Uniq_ratio** | **Total_tags** | **Total_ratio** |
| **rRNA** | 16636 | 70.74% | 822316 | 92.41% | 27043 | 56.86% | 779991 | 79.24% | 31111 | 61.86% | 2248404 | 92.83% |
| **tRNA** | 2047 | 8.70% | 21948 | 2.47% | 6590 | 13.86% | 131936 | 13.40% | 5822 | 11.58% | 99878 | 4.12% |
| **snoRNA** | 971 | 4.13% | 6366 | 0.72% | 3000 | 6.31% | 25707 | 2.61% | 2792 | 5.55% | 22210 | 0.92% |
| **snRNA** | 42 | 0.18% | 115 | 0.01% | 139 | 0.29% | 767 | 0.08% | 143 | 0.28% | 283 | 0.01% |
| **Other** | 3820 | 16.24% | 39153 | 4.40% | 10785 | 22.68% | 45930 | 4.67% | 10424 | 20.73% | 51335 | 2.12% |
| **Total** | 23516 | 100.00% | 889898 | 100.00% | 47557 | 100.00% | 984331 | 100.00% | 50292 | 100.00% | 2422110 | 100.00% |

|  | **LD-0h** | | | | **LD-8h** | | | | **LD-16h** | | | |
| --- | --- | --- | --- | --- | --- | --- | --- | --- | --- | --- | --- | --- |
| **RNA_Types** | **Uniq_tags** | **Uniq_ratio** | **Total_tags** | **Total_ratio** | **Uniq_tags** | **Uniq_ratio** | **Total_tags** | **Total_ratio** | **Uniq_tags** | **Uniq_ratio** | **Total_tags** | **Total_ratio** |
| **rRNA** | 13178 | 82.60% | 621479 | 95.83% | 53749 | 69.50% | 1799945 | 90.63% | 34871 | 62.21% | 1464258 | 87.59% |
| **tRNA** | 1010 | 6.33% | 10150 | 1.57% | 6589 | 8.52% | 97403 | 4.90% | 5401 | 9.64% | 74728 | 4.47% |
| **snoRNA** | 421 | 2.64% | 10558 | 1.63% | 3106 | 4.02% | 15534 | 0.78% | 3359 | 5.99% | 55394 | 3.31% |
| **snRNA** | 20 | 0.13% | 437 | 0.07% | 156 | 0.20% | 466 | 0.02% | 172 | 0.31% | 618 | 0.04% |
| **Other** | 1325 | 8.31% | 5916 | 0.91% | 13733 | 17.76% | 72716 | 3.66% | 12253 | 21.86% | 76809 | 4.59% |
| **Total** | 15954 | 100.00% | 648540 | 100.00% | 77333 | 100.00% | 1986064 | 100.00% | 56056 | 100.00% | 1671807 | 100.00% |
