# Supplementary material for: Identification of MicroRNAs in Response to Different Day Lengths in Soybean Using High-Throughput Sequencing and qRT-PCR
Source: PLoS One. 2015 Jul 10;10(7):e0132621. doi: 10.1371/journal.pone.0132621 (PMC4498749; doi:10.1371/journal.pone.0132621)
Supplement: S4 Table — The clean reads were compared with known miRNA precursors or mature miRNAs in the miRBase. The results of the six pools are shown. (DOCX) [file pone.0132621.s006.docx]

**S4 Table. Tags statistics of miRBase alignment. The clean reads were compared with known miRNA precursor or mature miRNA.**

| **Sample** | **Mature number** | **Hairpin number** | **Family number** | **Uniq_tag number** | **Total_tag number** |
| --- | --- | --- | --- | --- | --- |
| **Soybean_LD-0h** | 196 | 153 | 36 | 326 | 6907 |
| **Soybean_LD-8h** | 371 | 249 | 52 | 1048 | 60829 |
| **Soybean_LD-16h** | 342 | 233 | 50 | 892 | 62810 |
| **Soybean_SD-0h** | 152 | 118 | 29 | 201 | 1940 |
| **Soybean_SD-8h** | 269 | 197 | 45 | 590 | 27053 |
| **Soybean_SD-16h** | 376 | 247 | 52 | 1115 | 83338 |
