# Supplementary material for: Identification of MicroRNAs in Response to Different Day Lengths in Soybean Using High-Throughput Sequencing and qRT-PCR
Source: PLoS One. 2015 Jul 10;10(7):e0132621. doi: 10.1371/journal.pone.0132621 (PMC4498749; doi:10.1371/journal.pone.0132621)
Supplement: S6 Table — The novel predicted miRNAs were predicted through MIREAP. (DOCX) [file pone.0132621.s008.docx]

**S6 Table. Tags statistics of the novel miRNA. The novel miRNAs were predicted by MIREAP.**

| **Sample** | **New_miR_number** | **Unique tag** | **Total tag** |
| --- | --- | --- | --- |
| **Soybean_LD-0h** | 9 | 26 | 66 |
| **Soybean_LD-8h** | 22 | 138 | 632 |
| **Soybean_LD-16h** | 34 | 112 | 330 |
| **Soybean_SD-0h** | 4 | 24 | 47 |
| **Soybean_SD-8h** | 23 | 102 | 411 |
| **Soybean_SD-16h** | 24 | 191 | 633 |
