# Supplementary material for: Identification of MicroRNAs in Response to Different Day Lengths in Soybean Using High-Throughput Sequencing and qRT-PCR
Source: PLoS One. 2015 Jul 10;10(7):e0132621. doi: 10.1371/journal.pone.0132621 (PMC4498749; doi:10.1371/journal.pone.0132621)
Supplement: S9 Table — (DOCX) [file pone.0132621.s011.docx]

**S9 Table. The primer sequence of the miRNAs verified by qRT-PCR.**

| **Novel miRNA** | | **The primer sequence(5’to 3’)** |
| --- | --- | --- |
| **novel miRNAs** | **Soybean-miR-4** | AATGAGAACTGCGAAGGCCG |
|  | **soybean-miR-35** | AGTAGGCATGCGCTGATTGG |
|  | **soybean-miR-36** | CGGGGGGGAGTGAAATAGAACA |
|  | **Soybean-miR-57** | TATGGGGGGATTGGGAAGG |
|  | **Soybean-miR-60** | TCTTCCCTACACCTCCCATACC |
|  | **Soybean-miR-64** | GGGTGAGAACTTTGAAGGCCGA |
|  | **Soybean-miR-77** | GTCCGAGCCAGACTGTGATGTC |
| **conserved miRNAs** | **miR156a** | GCCCTGACAGAAGAGAGTGAGCA |
|  | **miR159e-3p** | GCGAGCTCCTTGAAGTCCAATT |
|  | **miR166a-3p** | TCGGACCAGGCTTCATTCC |
|  | **miR166a-5p** | GGAATGTTGTCTGGCTCGAGG |
|  | **miR482a-3p** | TCTTCTCAATTCTGCCCATTCC |
|  | **miR482c-3p** | TTCCCAATTCCGCCCATTAAG |
|  | **miR482a-5p** | GAGAATTTGTGGGAATGGGCTGA |
|  | **miR482b-5p** | GTATGGGGGGATTGGGAAGGA |
|  | **miR403a** | GCCCTTAGATTCACGCACAAACTT |
|  | **miR168a** | GAGAGCCACTTTTGGGTTCCCTAT |
|  | **miR390c** | CCCGCTATCCATCCTGAGTTTC |
|  | **miR167c** | GTGAAGCTGCCAGCATGATCTG |
|  | **miR396d** | GAAGAAAGCTGTGGGAGAATATGGC |
|  | **miR396b-3p** | GCTCAAGAAAGCTGTGGGAGAAAA |
|  | **miR396a-3p** | GGGGTTCAATAAAGCTGTGGGAAG |
|  | **miR2118b-3p** | TTGCCGATTCCACCCATTCCT |
|  | **miR2118b-5p** | GGAGATGGGAGGGTCGGTAAAG |
|  | **miR390a-3p** | CGCTGTCCGTCCTGAGTTTC |
|  | **miR160d** | TGCCTGGCTCTCTGTATGCC |
|  | **miR408b-3p** | CATGCACTGCCTCTTCCCTGG |
|  | **miR395a** | GCTGAAGTGTTTGGGGGAACTCA |
| **unconserved miRNAs** | **miR5371-5p** | TAGGAATGAGGCACTCGCG |
|  | **miR4412-5p** | GTGTTGCGGGTATCTTTGCCTC |
|  | **miR1507a** | GCCTCTCATTCCATACATCGTCTG |
|  | **miR1508b** | GGGTAGAAAGGGGAATAGCAGTTGA |
|  | **miR1508c** | GGGGTAGAAAGGGAAATAGCAGTTG |
|  | **miR1510a-3p** | GCTTGTTGTTTTACCTATTCCACCC |
|  | **miR1510b-3p** | GCCCTGTTGTTTTACCTATTCCACC |
|  | **miR1511** | GCGAACCAGGCTCTGATACCAT |
|  | **miR4998** | GCCAGTTTCGTGACTACAACTTCTG |
|  | **miR5032** | TTGGTGCAGGTCGGGAAAAA |
|  | **miR5368** | GGGGGGACAGTCTCAGGTAGACAA |
|  | **miR4413b** | GGGGGGCTAAGAGAATTGTAAGTCA |
