# Supplementary material for: Identification of MicroRNAs in Response to Different Day Lengths in Soybean Using High-Throughput Sequencing and qRT-PCR
Source: PLoS One. 2015 Jul 10;10(7):e0132621. doi: 10.1371/journal.pone.0132621 (PMC4498749; doi:10.1371/journal.pone.0132621)
Supplement: S10 Table — (DOCX) [file pone.0132621.s012.docx]

**S10 Table. The primer sequence of the targets verified by qRT-PCR.**

| **primer** | **Sequence(5’to 3’)** | **Ampification for Gene loci** | | **Targeted by miRNA** |
| --- | --- | --- | --- | --- |
| **1** | AAGGAAAGAGCAATGTGAAGCAG | *Glyma03g26830.1* | 159 | |
| **2** | GCCAGAGACAAAGTAAACCCCA |  |  | |
| **3** | CCACGCCAAAATGGGAAAC | *Glyma06g47000.1* | 159 | |
| **4** | CGCCTCTTCATCCGAGTGTT |  |  | |
| **5** | CGTGTCGTCTTCGTTGGGTCA | *Glyma07g14480.1* | 159 | |
| **6** | CCATCCTCTTTTGCCTACTGCTC |  |  | |
| **7** | AATGACTGGTATGGGGAAGGG | *Glyma12g03650.1* | 159 | |
| **8** | GATGTGGTACTCTGACTGAGTAGGC |  |  | |
| **9** | TGCTTGTGGCTGTGAGACTGG | *Glyma15g08630.1* | 159 | |
| **10** | ACTCTTGGAGGTTTTGATTGCTTC |  |  | |
| **11** | CAAACACTTGCTCGATGGATCT | *Glyma04g09000.1* | 166 | |
| **12** | AGAATGGACTCACTGCCTTCAC |  |  | |
| **13** | TTTTCTCCTGAGGACGTAGCG | *Glyma15g13640.1* | 166 | |
| **14** | TGCCCCAACTGCACTTTCA |  |  | |
| **15** | AACTGTGATGGTGCCGAGGAT | *Glyma12g08080.1* | 166 | |
| **16** | GCAGGAGGGACATTTTGAAGC |  |  | |
| **17** | GGGTGGACTGTATTGAACTGTGA | *Glyma11g20520.1* | 166 | |
| **18** | AATGTAAGGGAACTTGCTGGGT |  |  | |
| **19** | TAGGTTCTGGTAATGCTCGTCC | *Glyma15g13640.1* | 166 | |
| **20** | CCCGTGTATGGTTCTCAAAGG |  |  | |
| **21** | TATCATCGGTTCAAAGGGTAGG | *Glyma08g21620.1* | 166 | |
| **22** | GGGTTTGTGCTTCAGGAGTACC |  |  | |
| **23** | TCAACGCCAACATCAAAACC | *Glyma08g13110.1* | 166 | |
| **24** | CCAGTTGACAGCAGTTCCAGTAG |  |  | |
| **25** | ATGGCACGTCAATATGTTCGG | *Glyma07g01950.1* | 166 | |
| **26** | GCAAATCCAATGAGCAAGGGT |  |  | |
| **27** | GCAGAAGCAGGTCTCACAGTTG | *Glyma05g30000.1* | 166 | |
| **28** | TTTTGGGGTTTTGCGGTTG |  |  | |
| **29** | AAGAAAAGCGATGGGTGGTAA | *Glyma04g29220.1* | 1507 | |
| **30** | TCTCCCTTGGGCAGTCAGTC |  |  | |
| **31** | CAACACCTACACTGGAACTCAATG | *Glyma05g09220.1* | 1507 | |
| **32** | AGGAAGCCAAAGCAAAGACC |  |  | |
| **33** | GACAAGGTCGTAGCACAAGGATT | *Glyma09g07290.1* | 1508 | |
| **34** | TCAGCAACTTGACGGCACAT |  |  | |
| **35** | CAATGCCATAACCTTGACCACA | *Glyma09g30500.1* | 1508 | |
| **36** | GCCTAACCACTTGCCCTTCC |  |  | |
| **37** | AGAAGGAAAGGTGAAAGAAGCC | *Glyma16g28020.1* | 1508 | |
| **38** | TTGGAGCACAGCGTGGAAC |  |  | |
| **39** | CTACTGCCTAACCGTCTACATTCA | *Glyma04g03880.1* | 482 | |
| **40** | ATCATCCCCTGCTTCACCCT |  |  | |
| **41** | CTCCCCAAGTCACATTCTCGT | *Glyma17g07340.1* | 5032 | |
| **42** | TCTCGCAATCCATCATCCAC |  |  | |
| **43** | GCTTTTGCTCTCAGCTCTCATGG | *Glyma04g39740.1* | 1510 | |
| **44** | AGATTGGCGGCGAAACCCT |  |  | |
| **45** | CGTTAGTTGCTCGTGCTTGAC | *Glyma06g36310.1* | 1510 | |
| **46** | AAACCTTGGCAGTAAACCCTC |  |  | |
| **47** | TTCAAAAGGCAAGTAGGTTCGTG | *Glyma16g24940.1* | 1510 | |
| **48** | TCAGGGCAACCTCCAGCATAC |  |  | |
| **49** | GTAGAATGGGATGGGGATGC | *Glyma16g26270.1* | 1510 | |
| **50** | TTGGGACCTTCGGAAAACAG |  |  | |
| **51** | TGATGGCGATTGAAGAGTCC | *Glyma16g27540.1* | 1510 | |
| **52** | CAACCGACGCATTTCCTTAG |  |  | |
| **53** | GGAGATGCCCTCAAGAAGATG | *Glyma19g07660.1* | 1510 | |
| **54** | ACTGTTAGGAAAGTGTTTTGGACC |  |  | |
| **55** | GTGCTGGCATTTTCCTTTCG | *Glyma19g07680.1* | 1510 | |
| **56** | CTCCACCCTCATCCTCTTTACG |  |  | |
| **57** | TCAAAATGTGAGAAGGGTGAGC | *Glyma13g20370.1* | 160 | |
| **58** | GGAAGTGTCGGCAAAGGAAT |  |  | |
| **59** | GCCATTTCAGGTTGTTTACTATCC | *Glyma19g36570.1* | 160 | |
| **60** | TCCTCTGTTTCAAAGGGCATCT |  |  | |
| **61** | GGAGACAATACATCAATGCGGCT | *Glyma10g35480.1* | 160 | |
| **62** | GAAACACCTATCCAACAAAGGCAAC |  |  | |
| **63** | CCGTGGCTTGTTGAGTTGGTATC | *Glyma12g08110.1* | 160 | |
| **64** | GGGAAGTCTGGGTGTTGTGGC |  |  | |
| **65** | GCCACGGATGATGAGAATAAGG | *Glyma13g40030.1* | 160 | |
| **66** | GAAGAAAGGCAGGACAAGTCAAGT |  |  | |
| **67** | GAGTTCCGCCACATTTACCGC | *Glyma04g43350.1* | 160 | |
| **68** | ATCCTCATTCCGCCTCTATCACC |  |  | |
| **69** | GGAGCAACATCCCTTCTTTCCTAT | *Glyma13g02410.1* | 160 | |
| **70** | TGGTTTATCCATTTGTCCCACTG |  |  | |
| **71** | AAACGACACCACGGAGGCT | *Glyma14g33730.1* | 160 | |
| **72** | GCGGTAAATGTGGCGAAGC |  |  | |
| **73** | CTTGACAACATTCGTGAACCATTAG | *Glyma07g11290.1* | 4413 | |
| **74** | GCCTTCTTGACCTCTGACCCTTA |  |  | |
| **75** | AAGTTATTGGCACAAGGATTTCG | *Glyma07g11410.1* | 4413 | |
| **76** | GCAAGCCTCAGATACAAGTTTACG |  |  | |
| **77** | AATCGCTTACGCCCACCCT | *Glyma01g44420.1* | 4413 | |
| **78** | TCCATTGCCACATTCCACATC |  |  | |
| **79** | CAGATGGACCAGGTTAGTTACGC | *Glyma09g07250.1* | 4413 | |
| **80** | CCATTAACTGACCCGCAAGG |  |  | |
| **81** | TCATACCCCACCCATCATCC | *Glyma09g30940.1* | 4413 | |
| **82** | TGTTCAAAGTAGAAAGGTCAGGCT |  |  | |
| **83** | TGGACCAAGTCAGTTATGGGACG | *Glyma16g31950.1* | 4413 | |
| **84** | CTTCCTCAGTAATCTTGCGACAGC |  |  | |
| **85** | CGCCACCACCCAAACCTCTA | *Glyma11g01110.1* | 4413 | |
| **86** | CGCTTAACCTACCCCGGAACT |  |  | |
| **87** | GAATGTTAAGCGTTACCCCACTG | *Glyma14g38270.1* | 4413 | |
| **88** | AAGGCTAAGACCACTTGACCAAAG |  |  | |
| **89** | GCACGGGAGGTGTTTGATGAA | *Glyma15g24040.1* | 4413 | |
| **90** | CTCGTCTCGTCCACTTCGTTCTT |  |  | |
| **91** | ACTCAAGCAACCGTCAATCG | *Glyma20g37780.1* | 4413 | |
| **92** | AGCACCTAATACCAGTAGAAAATCG |  |  | |
| **93** | CAGAGTGGGTTGTTAAATGGAAAG | *Glyma18g51550.1* | 482c-3p | |
| **94** | CTGCCAAAGAAACTACCTCTAAATC |  |  | |
| **95** | CTATGCTGGGTCCTCATCCG | *Glyma16g00260.1* | 396d | |
| **96** | TGCGAGCAAAGAGGTAACAAAT |  |  | |
| **97** | TCAACTATGACTCAACGACCCAC | *Glyma06g12680.1* | 395a | |
| **98** | CTGAACTGAACACTTTAGCACCC |  |  | |
| **99** | AAGGGAAACATAGAAGACAACACC | *Glyma08g14700.1* | 395a | |
| **100** | TTATAAATCACTTGCCAACGAGG |  |  | |
| **101** | TGAGCCATCCAGTTGAGAAAAG | *Glyma10g38760.1* | 395a | |
| **102** | AGAATGTTTAGACGCTCTAGTCCC |  |  | |
| **103** | TTACACCGTTGGAGGACCTGG | *Glyma04g42120.1* | 395a | |
| **104** | TTCCCTGAACTGAACACTTTAGCAC |  |  | |
| **105** | CAGTCTTTCCTCGGAACATGC | *Glyma18g00890.1* | 156 | |
| **106** | TTTCTTTGCTGCTCCACCCT |  |  | |
| **107** | CCCTCTTCCTCCTCATCACCC | *Glyma02g30670.1* | 156 | |
| **108** | AGCCACCCCTTCCCTTCTTT |  |  | |
| **109** | CCATGTTTTCCAATGTTCGC | *Glyma18g36960.1* | 156 | |
| **110** | GCTCTTTCTTCTTCGCTCGTTA |  |  | |
| **111** | CCAAGGTGTCAAGTTGAAGGG | *Glyma19g32800.1* | 156 | |
| **112** | CCAGCAACAATGACTGTAGGG |  |  | |
| **113** | GATTTGGGTCACCACAGCATT | *Glyma05g00200.1* | 156 | |
| **114** | ATTGGGGCATCCACAACAGT |  |  | |
| **115** | AGAAGCGATGGGGTGAGATG | *Glyma05g38180.1* | 156 | |
| **116** | AGATTTCCAGGACTGAGCGG |  |  | |
| **117** | TGTCGAATTACCAAGGCACC | *Glyma06g17700.1* | 156 | |
| **118** | CACCACTCCAATCTGGGCTA |  |  | |
| **119** | AAAGCGGTGGATTGAGATGC | *Glyma08g01450.1* | 156 | |
| **120** | ATAAGTGGCGAGTGAGGGTTC |  |  | |
| **121** | TGGAAGATCCCATTAGGTTGTC | *Glyma05g38180.1* | 156 | |
| **122** | GCCGAGGAAGGAAGTGGTAAGT |  |  | |
| **123** | TGCCAAAACTGGGGCTGAAT | *Glyma17g08840.1* | 156 | |
| **124** | GGCGAGGGTGCCTTGAATAG |  |  | |
| **125** | GGACACTGTAGGAAATGAGGACTT | *Glyma05g00200.1* | 156 | |
| **126** | TAGGGATGGCTATGACTGCTTG |  |  | |
| **127** | GCAGCCATCCATCAACTTCC | *Glyma16g05900.1* | 156 | |
| **128** | CACCCTCAGGCAAACAATCAC |  |  | |
| **129** | CGAAAAGCACTCCAAAACCC | *Glyma19g26390.1* | 156 | |
| **130** | TACTTGAACTTCTCAGCAGCCAT |  |  | |
